# Does technique matter; a pilot study exploring weighting techniques for a multi-criteria decision support framework

**DOI:** 10.1186/1478-7547-12-22

**Published:** 2014-11-18

**Authors:** Janine van Til, Catharina Groothuis-Oudshoorn, Marijke Lieferink, James Dolan, Mireille Goetghebeur

**Affiliations:** University of Twente, MB-HTSR, PO Box 217, 7500 AE Enschede, The Netherlands; University of Rochester, Rochester, NY USA; LASER Analytica & University of Montreal, Montreal, Canada

**Keywords:** Multi-criteria decision analysis, Preferences, Weighting techniques, Decision support, Health care

## Abstract

**Background:**

There is an increased interest in the use of multi-criteria decision analysis (MCDA) to support regulatory and reimbursement decision making. The EVIDEM framework was developed to provide pragmatic multi-criteria decision support in health care, to estimate the value of healthcare interventions, and to aid in priority-setting. The objectives of this study were to test 1) the influence of different weighting techniques on the overall outcome of an MCDA exercise, 2) the discriminative power in weighting different criteria of such techniques, and 3) whether different techniques result in similar weights in weighting the criteria set proposed by the EVIDEM framework.

**Methods:**

A sample of 60 Dutch and Canadian students participated in the study. Each student used an online survey to provide weights for 14 criteria with two different techniques: a five-point rating scale and one of the following techniques selected randomly: ranking, point allocation, pairwise comparison and best worst scaling.

**Results:**

The results of this study indicate that there is no effect of differences in weights on value estimates at the group level. On an individual level, considerable differences in criteria weights and rank order occur as a result of the weight elicitation method used, and the ability of different techniques to discriminate in criteria importance. Of the five techniques tested, the pair-wise comparison of criteria has the highest ability to discriminate in weights when fourteen criteria are compared.

**Conclusions:**

When weights are intended to support group decisions, the choice of elicitation technique has negligible impact on criteria weights and the overall value of an innovation. However, when weights are used to support individual decisions, the choice of elicitation technique influences outcome and studies that use dissimilar techniques cannot be easily compared. Weight elicitation through pairwise comparison of criteria is preferred when taking into account its superior ability to discriminate between criteria and respondents’ preferences.

**Electronic supplementary material:**

The online version of this article (doi:10.1186/1478-7547-12-22) contains supplementary material, which is available to authorized users.

## Background

Regulatory authorities face a complex decision task when considering new drugs or medical technologies for access and reimbursement. They need to take into account multiple aspects of a new technology and weigh their impact on the value of the innovation. Important aspects include: evidence on clinical benefits and risks, effects on quality of life, costs to the health care system, severity of disease, the context of use and equity [1]. On a lower level, local health care decision makers such as hospital managers face similar tasks; although, the criteria on which decision are made may differ.

Because these decisions are complex if made unaided, decision makers typically use heuristic or intuitive approaches to simplify them [2]. Thus, the quality of regulatory and reimbursement decisions is under scrutiny and decisions made by these authorities are regularly criticized for their lack of repeatability, validity and transparency [3].

In recent years, multiple initiatives were undertaken to improve the decision making process in the policy decision making setting. Multi-Criteria Decision Analysis (MCDA) was proposed as a way to overcome some of these problems [4]. MCDA, an application of analytical methods to explicitly consider multiple criteria, is used as an umbrella term to describe a range of different methods. MCDA methods rely on several steps before they identify the value of the decision alternatives, such as defining the problem, determining goals and requirements to the analysis, selecting criteria, determining their hierarchy, weighting the criteria and scoring the performance of alternative solutions to the problem [5].

In an extensive project coordinated by the European Medicines Agency (EMA)(IMI-PROTECT), the use of multi-criteria decision analysis (MCDA) has been proposed as an approach to perform quantitative risk-benefit modeling and help decision-makers construct the values that are fundamental to effective decisions [6]. Recently, Devlin and Sussex argue for greater use of MCDA as an aid to decision making by national health services (NHS) in the UK [7].

The actual use of MCDA techniques to support decisions in health care is increasing [8]. One such example is the MCDA framework used to support coverage decisions by the Ontario Health Technology Advisory Committee (OHTAC) in Canada [9]. The EVIDEM framework was developed as an open source comprehensive framework that aims to connect the principles of MCDA and of health technology assessment (HTA) [10]. In recent years, the EVIDEM framework has been further developed, tested, and adapted in several jurisdictions and contexts [9, 11–13]. A process combining the EVIDEM framework with the EUNEtHTA Core model has been successfully implemented by the Lombardy Health Region in Italy for HTA and coverage decisions [14]. The most recent version proposes fourteen core quantitative criteria and seven contextual criteria which can be used to evaluate the value of healthcare interventions in different decision contexts.

Building on an open source philosophy, the EVIDEM framework is collaboratively developed and regularly updated based on the practical experience and methodological issues identified in pilot testing and implementation. Important areas for further study that were identified were: the decision to use the five point rating scale to weight criteria, whether or not a hierarchy of decision criteria should be included to simplify weighting, and whether the choice of the weight elicitation technique had an impact on the measured value of healthcare interventions. While some other MCDA methods include very specific recommendations on the weight elicitation technique and put specific demands on the hierarchy of criteria, the EVIDEM framework aims to offer a more flexible approach [15, 16].

The mixing and matching of weight elicitation techniques to meet the specific demands of the decision context is common practice. Criteria for selecting a weighting technique include: perceived face validity in the decision context, cognitive effort required from the respondent, and difficulty in analysis. Although these issues are important, the first question to be answered is whether choice of method would influence the validity of the results of the analysis and its overall impact on the outcome of the MCDA exercise. The fact that there is no golden standard for weighting, i.e. no measure of a “true” weight is available, challenges validity testing of weighting techniques. In this study we assume that the main aim of weighting in MCDA is to estimate the respondent’s priorities in satisfying different decision criteria. Therefore, an important aspect in the choice of weighting technique is its ability to differentiate between criteria that are considered important and those which are not. The objectives of this study were to test: 1) the influence of weights elicited with different techniques on the overall outcomes of a multi criteria decision analysis, 2) whether different weight elicitation techniques have equal discriminative power in weighting different criteria, and 3) whether different techniques result in similar weights in weighting the criteria set proposed by the EVIDEM framework.

## Methods

### Survey design

To familiarize respondents with the decision context, a health care decision problem was presented. Respondents were asked to take the perspective of a health policy maker having to decide on the reimbursement of one of multiple health innovations, with limited funds available.

Then respondents were introduced to the concept of multiple criteria decision making: *“in making the decision, you have to take into account multiple criteria. The ability of the health innovations to satisfy these criteria differs, and sometimes these criteria are conflicting (i.e. small benefits for many people or large benefits for few). Therefore it is important to know how you prioritize these criteria in your decision*.” After the decision problem was introduced, respondents were asked to indicate, for 14 different criteria, their relative importance in deciding on reimbursement of a health innovation from a societal perspective (Appendix A).

There are many weighting techniques in MCDA. We selected four different weight elicitation techniques based on diversity in formatting and their frequency of use [17]:

1. The five-point rating exercise is a technique in which all criteria are rated on a five-point scale (RS) ranging from (1) not important to (5) very important. The five-point RS was the initial technique proposed in the EVIDEM framework [10].
2. The ranking (RA) exercise is a technique in which criteria are ranked from most important to least important. Ranking is a commonly used method to prioritize criteria in simple-multi-attribute utility theory (SMART) and often combined with point allocation (PA) where a budget of 100 points is allocated over criteria to reflect their relative importance [18–20].
3. The pairwise comparison (PC) technique compares criteria on a reciprocal numerical rating scale ranging from 9 (strong preference for criteria A) to 9 (strong preference for criteria B). If criteria are considered equally important, a score of 1 is given. This technique is used in the analytic hierarchy process (AHP) [21].
4. In best worst scaling (BWS), subsets of four criteria are presented, and respondents are asked to select the most important and least important from the set. This is performed 12 times and all criteria are presented equally often. This method is known as best worst scaling (BWS) case 1 [22, 23].

**Figure 1:**
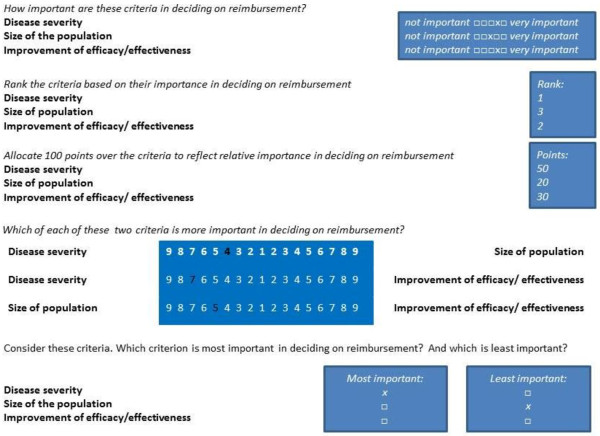
**Example of the weight elicitation technique in the survey.**

Ranking, rating and point allocation are direct methods to assess criteria importance. For each criterion, one value judgment is required (Figure 1). In contrast, in pairwise comparison and in BWS, multiple relative judgments are required for each criterion. In pairwise comparison, the number of judgments required to compare 14 criteria is 91. Because this was considered too time-demanding, the pairwise comparisons criteria were ordered hierarchically based on their category as proposed in the EVIDEM framework (Additional file 1: Figure S1). The same hierarchical decision tree was used for ranking and point allocation because ranking 14 criteria and allocating 100 points over these criteria was considered too cognitively demanding for respondents.

To study the effect of introducing this variation in the hierarchical ordering of criteria, the five-point rating scale, which easily accommodates both hierarchical as well as nonhierarchical ordering, was used to elicit preferences in both conditions. This resulted in five different ways to estimate criteria weights.

Respondents were asked to use the five-point rating scale (RS_nH) and either: (1) ranking (RA) and rating through point allocation (PA) (n = 15), (2) pairwise comparisons (PC; n = 15), (3) best worst scaling (BWS; n = 15) or (4) the five point rating scale with criteria ordered in a hierarchical structure (RS_H; n = 15) to elicit criteria weights to state the perceived importance of the 14 criteria. The order of the five point rating scale and the other technique was randomly varied among respondents. After using both techniques, respondents were asked which of the two techniques they preferred. At the end of the survey, respondents were asked to provide some background characteristics.

### Respondent sample

This study was conducted at the University of Twente in the Netherlands and the University of Montreal, Canada. The study was approved by the Board of Ethics of the University of Montreal and exempted from ethical approval in the Netherlands. Students from the health oriented courses at both universities were invited to participate in the study via mass e-mail sent to the university student accounts of the studies health sciences, biomedical engineering and technical medicine. At the time of the study about 200 students attended the courses that were targeted. Exactly how many students were reached is unknown, as the e-mail was sent by university personnel to ensure privacy of the students. After three weeks, a reminder was sent.

### Data analysis

Enrollment in this pilot study was stopped on pragmatic grounds (limited time and funding available) after 60 students completed the survey. All the data was analyzed with PASW Statistics 18 and Stata 12.0. Criteria weights were calculated for each technique and descriptive analysis (mean, standard deviation) was performed.

The influence of criteria weights on the overall value estimate of interventions was explored by calculating the overall value (V) that would result from multiplying the normalized criteria weights (*w*_*i*_) obtained in this study with performance scores (*v*_*i*_) obtained in a previous study [12]. The overall value estimate was calculated using the equation:


To compare the value estimates, they were normalized into a score out of 100 by dividing the overall expected value of the alternative by the maximum value score.

For presentation purposes, the rank order of the 14 criteria was calculated based on the mean normalized criteria weights for each method (Table 1). Rank reversals between the five-point rating scale and the other method are calculated on a group level and the individual level where the rank order based on five-point rating scale are used as the reference ranking.

**Table 1:** **The effect of weighting technique on rank order of criteria and weight differences between criteria**

Characteristics	Five point rating scale (n = 60)		Five point rating (hierarchical) (n = 15)		Point allocation (hierarchical) (n = 15)		Ranking (n = 15)		Pairwise comparison (hierarchical) (n = 15)		Best worst scaling (n = 15)	
Criteria rank order (mean group weight)												
1st	D2	0.100	D1	0.087	D1	0.092	D1	0.095	D2	0.118	D2	0.091
2nd	D1	0.089	Q1	0.086	I3	0.089	C2	0.088	D1	0.111	I1	0.090
3rd	I3	0.085	T1	0.083	D2	0.084	I3	0.086	I3	0.110	D1	0.089
4th	I1	0.080	D2	0.082	C2	0.082	Q1	0.083	C2	0.083	I3	0.083
5th	Q2	0.076	Q2	0.077	Q1	0.078	D2	0.080	T1	0.082	T1	0.078
6th	T1	0.075	C2	0.076	I1	0.077	I1	0.078	I1	0.071	E1	0.077
7th	C2	0.072	T2	0.070	E3	0.070	E3	0.077	Q2	0.060	E2	0.074
8th	Q1	0.069	I3	0.070	E2	0.068	Q2	0.069	I2	0.056	I2	0.073
9th	E2	0.066	I1	0.067	Q2	0.065	E2	0.065	Q1	0.056	E3	0.069
10th	I2	0.065	C1	0.063	E1	0.062	E1	0.063	C1	0.054	C2	0.064
11th	E1	0.061	E2	0.062	I2	0.060	C1	0.056	E1	0.054	Q1	0.057
12th	E3	0.057	E1	0.062	T1	0.059	I2	0.055	T2	0.052	T2	0.056
13th	T2	0.057	E3	0.061	T2	0.058	T1	0.053	E2	0.049	Q2	0.054
14th	C1	0.052	I2	0.053	C1	0.056	T2	0.053	E3	0.044	C1	0.046
Weight difference												
1st-3rd^*^	0.027 (0.04)		0.009 (0.01)		0.030 (0.03)		0.034 (0.03)		0.075 (0.10)		0.017 (0.02)	
Most-least^**^	0.091 (0.07)		0.064 (0.02)		0.100 (0.05)		0.110 (0.05)		0.156 (0.12)		0.076 (0.02)	
Equal weights^***^	0.022 (0.01)		0.017 (0.01)		0.027 (0.01)		0.027 (0.01)		0.034 (0.02)		0.018 (0.01)	
Rank difference												
Group ranks	#		2.8 (2.8)		2.0 (1.8)		2.5 (2.1)		1.6 (1.4)		2.3 (2.1)	
Individual ranks	#		7.5 (1.7)		7.6 (2.6)		7.8 (3.5)		8.3 (1.9)		6.2 (2.5)	
Correlation												
Mean across-respondents	##		0.83		0.83		0.71		0.78		0.97	
Mean within-respondents	##		0.53		0.50		0.43		0.45		0.66	

^*^Within respondent difference between the weight of the first and third ranked criteria (group mean and standard deviation). ^**^Within respondent difference between the weight of the first and last ranked criteria (group mean and standard deviation). ^***^Within respondent difference between weights elicited with the technique and equal weights for all criteria (mean and standard deviation). # rank order based on five point weighting scale (nH) criteria ordering is the reference ranking. ## weights of five point rating scale (nH) is the reference weight. Criteria abbreviations: D1: Disease severity, D2: Size of population, I1: Improvement of efficacy/effectiveness, I2: Improvement of safety/tolerability, I3: Improvement of patient-reported outcomes, Q1: Completeness and consistency of reporting evidence, Q2: Relevance and validity of evidence, C1: Clinical guidelines, C2: Comparative interventions limitations, T1: Public health interest, T2: Type of medical service, E1: Budget impact on health plan, E2: Cost-effectiveness of intervention, E3: Impact on other spending.

We used three measures to study discriminative power (ability of a technique to prioritize criteria) of the weighting techniques: 1) the mean of the weight distance between the 1st and 3rd most important criterion within each respondent for all methods; 2) the mean of the weight distance between most and least important criterion within each respondent for all methods; and 3) the mean of the weight distance between the weight for each criterion and the weight for each criterion if no method was used to prioritize criteria (equal weights for 14 criteria = 0.07). To test whether the techniques enable prioritization in criteria based on the weights which are elicited with the different techniques, we used a one sample t-test to evaluate whether the difference in weights between the 1st and 3rd and the most and least important criterion were significantly different from zero. If no prioritization of weights occurs, all weights would be equal (thus the difference between the most and least important weight were zero).

We used the correlation of the across-respondents means and the mean within-respondent correlation to evaluate whether weights elicited with different methods resulted in similar weights, as proposed by Nickerson [24]. To visualize the agreement between the weights elicited with different techniques, Bland Altman Plots were used [25, 26] ^a^. First, we plotted the weights per criteria averaged over all respondents that had the same combination irrespective of the order of weighting techniques against the difference between these mean weights per criteria separately for each second weighting technique. Second, we plotted the same outcomes but then for each respondent individually. These Bland-Altman plots allowed us to study any systematic difference between the elicitation methods (i.e., fixed bias) on a group level and individually.

## Results

### Respondents

Of the 200 students invited to participate in the study, 88 started the online survey. 18 respondents did not finish the survey, and their results were excluded from the analysis. Background characteristics are not available for respondents that did not complete the survey. The sample was over representative of females, younger and highly educated individuals compared to the average population (Table 2).

**Table 2:** **Respondent sample background characteristics**

Characteristics	
Gender	
Male	11 (18%)
Female	49 (82%)
Age	
18-22	22 (37%)
23-30	37 (62%)
41 or older	1 (1%)
Education	
Bachelor	44 (73%)
Master	16 (27%)

### Criteria importance

Table 1 reports the rank order of the criteria across the different methods based on their mean weight (group level data). Disease severity (D1) was always ranked among the three most important criteria and was ranked as most important with three out of six methods. Size of the population (D2) was ranked in the top 3 with four out of six methods. In total, seven different criteria were ranked among the top 3 including: Disease severity (D1) (6 times), Size of population (D2) (4 times), Improvement of patient reported outcomes (I3) (4 times), Comparative intervention limitations (C2) (1 time), Improvement of efficacy (I1) (1 time), Public health interest (T1) (1 time), and Quality of evidence (Q1) (1 time). In the bottom three, eight criteria were represented. Most often ranked in the bottom three were Clinical guidelines (C1) (3 times), Impact on other spending (E3) (3 times) and Type of medical service (T2) (5 times). Based on criteria weights, criteria can be ranked. The effect of weighting technique on the difference in ranking of a criterion on an individual level is much higher (difference in rank between 6 and 8 ranks, depending on method) than a difference in ranking of a criterion on a group level (about 2 ranks difference for each method).

### Impact on overall value estimate

The differences observed in the ability of weighting techniques to prioritize criteria did not result in large differences in value estimates on a group level. Mean value estimates fluctuate around 46% of the maximum value, independent of the method used to elicit weights (Table 3). These overall value estimates are not significantly different from each other (p = 0.75). However, at the individual level, there are considerable differences in value estimates. With best-worst scaling, the overall value estimate varies between 42% and 47%. With pair-wise comparisons, the overall value estimates on an individual level vary between 38% and 59% of the maximum value. Hierarchical ordering of the decision tree reduces the range of value estimates within respondents from 15% to 5%.

**Table 3:** **Mean and distribution of overall value estimates**

Method	N	V (standard error; standard deviation)		% of maximum V [min-max]	
Five point rating (nH)	75	1.370	(0.004; 0.065)	45.7	[40–55]
Ranking	15	1.362	(0.004; 0.065)	45.4	[42–49]
Point allocation	15	1.364	(0.006; 0.075)	45.5	[42–51]
Pairwise comparison	15	1.397	(0.027; 0.166)	46.6	[38–59]
Best-worst scaling	15	1.362	(0.002; 0.039)	45.4	[43–47]
Five point rating (H)	15	1.356	(0.002; 0.042)	45.2	[42–47]

### Ability of each method to discriminate based on criteria importance

As is demonstrated in Table 1 the absolute difference in weight of the most important and least important criterion based on group average varied between 0.034 and 0.074. For instance, the difference in weight between most (d2 = 0.100) and least important (C1 = 0.052) for five point rating scale is 0.048.

Individual weights elicited with pair-wise comparison have the highest absolute difference in weight between: the three most important criteria (0.075), the most and least important criteria (0.156) and the average absolute difference between weighted criteria importance, and equal weights (0.034). These differences are smallest with five point rating scale in a hierarchical ordering. For all techniques, the difference in weights between most and least important criteria are significantly different from zero (equal weighting) on an individual level, as is the difference in weights of the first and third ranked criterion (p < 0.05). This indicates that all techniques enable significant discrimination in criteria importance compared to no weighting of criteria.

### Agreement between criteria weights elicited with different elicitation techniques

**Figure 2:**
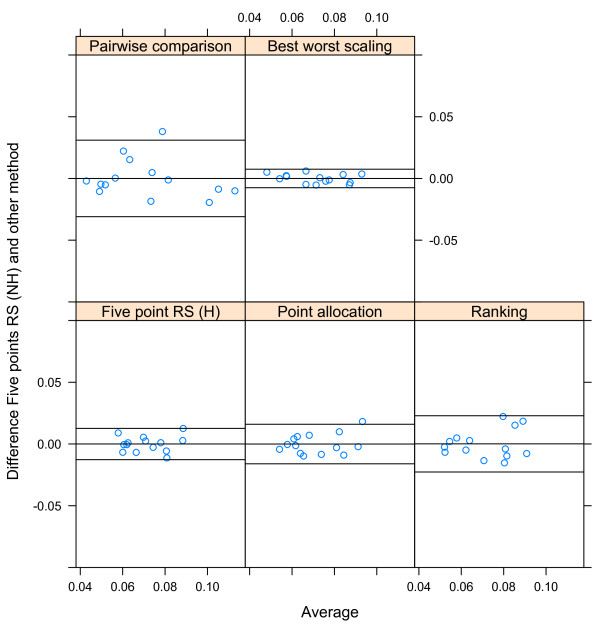
**The effect of weight elicitation technique on criteria weight estimates (Bland-Altman Plot).**

The results of this study indicate that agreement across groups of respondents using the same techniques is high. The highest across respondent correlation was found between five point rating scale and best worst scaling (0.97), and the lowest correlation is found between five point rating scale and ranking (0.71) (Table 1). From the Bland Altman plot presented in Figure 2, it can be concluded that there is no systematic bias on the group level when the five point rating scale is compared to the other techniques. Almost all differences in group weights are within the limits of agreement. This indicates that, when averaged on a group level, the effect of the weighting technique on the ability to give consistent weights is small.

However, the agreement within respondents’ weights was found to be moderate (between 0.42 and 0.66). This indicates that the ability of an individual to give consistent weights with different techniques is lower and is dependent on the combination of techniques.

### The stability of the preferences elicited with different methods based on order in the survey

**Figure 3:**
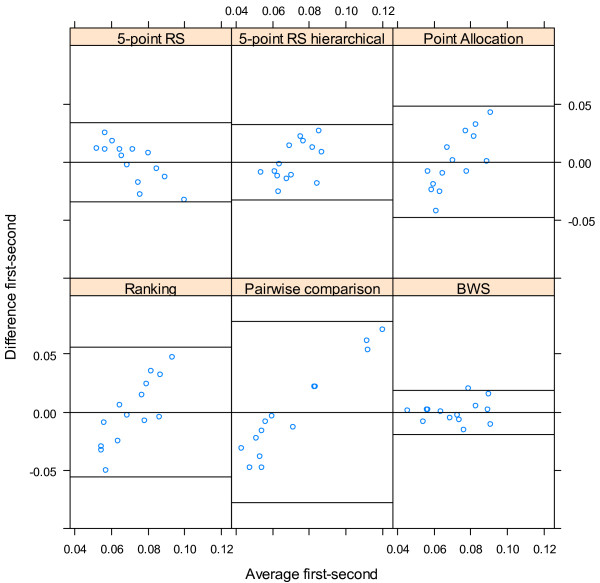
**The effect of order in the survey on criteria weight estimates (Bland-Altman Plot).**

If preferences are stable and are not influenced by the experience of the respondent, for instance with weight elicitation or familiarity with the decision context, the order in which weighting techniques were presented in the survey would not result in a systematic bias in the criteria weights elicited with that technique. Figure 3 demonstrates that the differences in group mean weights elicited with the same method are influenced by the order in the survey. For point allocation, ranking and pairwise comparisons, the results demonstrate that when the technique is used first, respondents ability or willingness to discriminate between criteria based on their importance is higher. If these techniques are used after another weight elicitation technique, the lower ranked criteria are weighted higher and the higher ranked criteria are weighted lower, which indicates an anchoring effect. For these techniques, experience seems to result in a tendency to discriminate less between criteria. BWS shows no consistent relationship between order in the survey and difference in weights for the criteria.

### Respondent preferences

Respondents were also asked which technique they preferred. The majority of respondents (74%) preferred pair-wise comparisons or ranking combined with point allocation (69% of the respondents) to the five point rating scale. 87% of respondents preferred five point rating scale over best-worst scaling. Hierarchical weighting with five point rating scale is preferred (66%) to non-hierarchical weighting.

## Discussion

The aim of weighting in MCDA is to quantitatively reflect the relative importance of multiple criteria in order to value healthcare intervention. Earlier research has shown that decision makers choose techniques based on their fit to the decision context, and cognitive and time demands in collecting and analyzing their results [27]. Given the diversity of MCDA methods available the selection of the weighting technique requires insight in the specific strengths and weaknesses of the different methods. The objectives of this study were: to assess the effect of five different weighting techniques on the value of health innovations, to compare their ability to discriminate between criteria, and to test whether these different techniques result in similar weights.

The first objective of this study was to test the influence of weights elicited with different techniques on the overall outcome of the decision analysis. In agreement with earlier studies, in this study mean differences in outcome are negligible at the group level. However, the overall value of the innovation was judged differently on the individual level with pairwise comparisons and five-point rating scale showing the largest difference in overall value estimates between individuals.

The second objective of this study was to compare the ability of techniques to discriminate between criteria which is reflected in the range in weights between different criteria. Weight range is important because it reflects the ability of the weighting technique to distinguish between criteria of high and low importance and thus to prioritize outcomes in estimating the value of health innovations [28]. Absolute differences in weights were small. However, this is a result of scaling effects as the weights add to one. The weight given to the most important criterion is significantly different from the criterion ranked third with all techniques. Thus, all methods are able to relatively discriminate between criteria based on their perceived importance. However, the extent to which they are able to do so is different. Pairwise comparisons of criteria have the highest discriminative ability. This is partly explained by the elicitation scale since 5-point single scale allows for less diversity in responses than a 9-point reciprocal scale. More choice for the respondents could result in higher cognitive demands on the respondents, but the finding that pairwise comparisons were preferred to the other techniques by the majority of the respondents indicates that this is not considered a burden in this highly educated sample.

The third objective of this study was to test whether the choice for a different weighting technique would result in different weight estimates for the criteria. Across respondents, weights elicited with different techniques have high correlation, indicating they measure the same underlying perceived importance. Across respondents, mean comparability of weights elicited with different techniques is relevant if the intended application involves value means as in program evaluation and decision analysis. However, when weights elicited within one respondent with different techniques are compared, correlation is only moderate. Mean within respondent comparability of weights is important if the intended application requires individual level values as in clinical decision making.

In the practical context, the results of this study have several implications. The EVIDEM framework considers 14 core criteria. This reflects the reality of health care decisions at the local, national or even global level are influenced by many criteria [1, 2, 29, 30]. All techniques accommodate weighting of this many criteria; although some need adaptations to the ordering of criteria. However, in further development of the EVIDEM framework, developers might consider using pairwise comparisons of criteria if higher discrimination of estimates of weight and overall outcome is desired. This might be the case if the decision is made on an individual level or if differences between decision makers are considered important in the decision. A barrier for the use of pairwise comparisons is the steep increase in the number of comparisons required if a large number of criteria are being considered on the same level of the decision. The use of partial profile experimental designs might be considered to reduce this barrier [31]. Although all methods result in significantly different weight estimates for the criteria, absolute differences in weights presented on the 0–1 scale are small. This might hinder discernibility of the differences to the decision maker, especially when 14 criteria are considered. If the calculation of overall value is shared with decision makers who are not familiar with MCDA, it is suggested to change the format of the weights, for example, by multiplying them with 100. This effect of scaling would not influence the outcome of the analysis, but increases the obviousness of differences in the criteria weights.

### Limitations

There are some limitations that should be taken into account when interpreting the results of this study. There was an effect of order of the weight elicitation technique in the survey. With all techniques, except for combinations that include best-worst scaling, respondents discriminate less the second time they are asked to provide weights. One reason could be that preferences are constructed during the elicitation process [32]. A study that focused on the influence of stability of preferences during weight elicitation, and especially how much time or experience is required for preferences to become stable is recommended. In this regard, it must be taken into account that the respondents were highly educated university bachelor students in health sciences, biomedical engineering or medicine, that are most likely unfamiliar with the considerations in policy decision making. This might have influenced the stability of their preferences. Actual decision makers, especially if they have gained experience with MCDA and weight elicitation, might have more stable views on criteria importance, as observed in a previous study involving experienced members of a standing decision making committee [9]. Other limitations to our study include the relatively high number of criteria, its small sample size and the possible influence of survey formatting (use of an online survey, the order in which the criteria were presented) on the outcome of the study. Further research is recommended in a larger sample of respondents familiar with the decision context, and to randomize not only the order in which different techniques are presented, the number of criteria which are compared but also the order of the criteria.

## Conclusions

For MCDA to gain wider use as a decision support tool in regulatory and reimbursement decision making clear guidance on the choice of techniques is needed. Although uniformity in techniques would be beneficial, there are many issues to consider in choosing a weighting technique, such as theoretical foundation, its fit to the decision context and its efficiency in collecting and analyzing preferences. The results of this study show that based on its ability to discriminate between criteria weights in a decision setting with many criteria, pairwise comparison is the preferred technique to elicit weights. However, the results of this study also show that on an aggregate level, the differences in weight as a result of technique have limited impact on the overall outcome of the analysis, and thus the choice of weighting technique has limited impact on the comparability of results of MCDA in a group setting.

Alternatively, if the aim of the analysis is to compare individual preferences, for instance of different members within decision panels, the results of this study indicate the use of different weight elicitation techniques will result in considerable differences in weight estimates and outcome of the analysis, making uniformity in weight elicitation technique more important.

## Endnote

^a^A Bland-Altman plot, also known as a Tukey Mean difference plot, is a well-recognized method to study agreement between measurements. In a Bland-Altman plot the mean of two measurements on the same object is plotted against the difference between these two measurements (this is the relative bias). If the points of this plot are scattered randomly above and below the x-axis, then there is no consistent bias of one measurement over the other. If the difference between the measurements deviates from zero there is no agreement between the measurements. A significant bias can be shown with a paired *t*-test. Moreover, the standard error of these differences represents the magnitude of error between the two measurements. If the differences between the two measurements are not clinically important, the two methods may be used interchangeably. Points outside the limits of agreement (95% confidence intervals of the differences) are considered outliers. A linear trend of the differences for higher values or lower values indicates that one method overestimates high values and under-estimates low values.

## Appendix A: List of criteria of the EVIDEM framework and definitions

### Disease impact

D1: **Disease severity**: Severity of the health condition of patients treated with the proposed intervention (or severity of the health condition that is to be prevented) with respect to mortality, disability, impact on quality of life, clinical course (i.e., acuteness, clinical stages).

D2: **Size of population**: Number of people affected by the condition (treated or prevented by the proposed intervention) among a specified population at a specified time; can be expressed as annual number of new cases (annual incidence) and/or proportion of the population affected at a certain point of time (prevalence).

### Context of intervention

C1: **Clinical guidelines**: Concurrence of the proposed intervention (or similar alternatives) with the current consensus of a group of experts on what constitutes state-of-the-art practices in the management of the targeted health condition; guidelines are usually developed via an explicit process and are intended to improve clinical practice.

C2: **Comparative interventions limitations** (unmet needs): Shortcomings of comparative interventions in their ability to prevent, cure, or ameliorate the condition targeted; also includes shortcomings with respect to safety, patient reported outcomes and convenience.

### Intervention outcomes

I1: **Improvement of efficacy/effectiveness**: Capacity of the proposed intervention to produce a desired (beneficial) change in signs, symptoms or course of the targeted condition above and beyond beneficial changes produced by alternative interventions.

I2: **Improvement of safety & tolerability:** Reduction in intervention-related health effects that are harmful or undesired compared to alternative interventions.

I3: **Improvement of patient reported outcomes**: Capacity of the proposed intervention to produce beneficial changes in patient-reported outcomes (PROs) (e.g., quality of life) above and beyond beneficial changes produced by alternative interventions; also includes improvement in convenience to patients and adherence to treatment course.

### Type of benefit

T1: **Public health interest** (e.g., prevention, risk reduction): Risk reduction provided by the proposed intervention at the population-level (e.g., prevention, reduction in disease transmission, reduction in the prevalence of risk factors).

T2: **Type of medical service** (e.g., cure, symptom relief): Nature of the clinical benefit provided by the proposed intervention at the patient-level (e.g., symptom relief, prolonging life, cure).

### Economics

E1: **Budget impact on health plan** (cost of intervention): Net impact of covering the intervention on the budget of the target health plan (excluding other spending, see impact on other spending). This represents the differential between expected expenditure for the proposed intervention and potential cost savings that may result from replacement of other intervention(s) currently covered by the health plan. Limited to cost of intervention (e.g. acquisition cost, implementation cost).

E2: **Cost-effectiveness of intervention**: Ratio of the incremental cost of the proposed intervention to its incremental benefit compared to alternatives. Benefit can be expressed as number of events avoided, life-years gained, quality-adjusted life-years gained, additional pain-free days etc.

E3: **Impact on other spending** (e.g., hospitalization, disability): Impact of providing coverage for the proposed intervention on other expenditures (excluding intervention cost, see budget impact on health plan) such as hospitalization, specialist consultations, adverse events, long-term care, disability costs, lost productivity, caregiver time, equipment maintenance cost etc.

### Quality of evidence

Q1: **Completeness and consistency of reporting evidence**: Extent to which reporting of evidence on the proposed intervention is complete (i.e., meeting scientific standards on reporting) and consistent with the sources cited.

Q2: **Relevance and validity of evidence**: Extent to which evidence on the proposed intervention is relevant to the decisionmaking body (in terms of population, disease stage, comparator interventions, outcomes etc.) and valid with respect to scientific standards (i.e., study design etc.) and conclusions (agreement of results between studies). This includes consideration of uncertainty (e.g., conflicting results across studies, limited number of studies & patients).

## Electronic supplementary material

Additional file 1: Figure S1: Hierarchy of criteria. (DOC 180 KB)
