# Supplementary material for: Does technique matter; a pilot study exploring weighting techniques for a multi-criteria decision support framework
Source: Cost Eff Resour Alloc. 2014 Nov 18;12:22. doi: 10.1186/1478-7547-12-22 (PMC4406027; doi:10.1186/1478-7547-12-22)
Supplement: Supplementary file 1 — Additional file 1: Figure S1: Hierarchy of criteria. (DOC 180 KB) [file 12962_2014_204_MOESM1_ESM.doc]

Additional file 1: Figure S1. Hierarchy of criteria


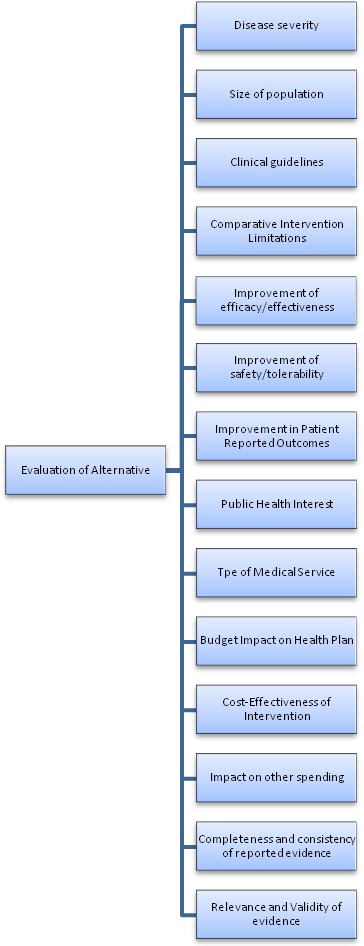

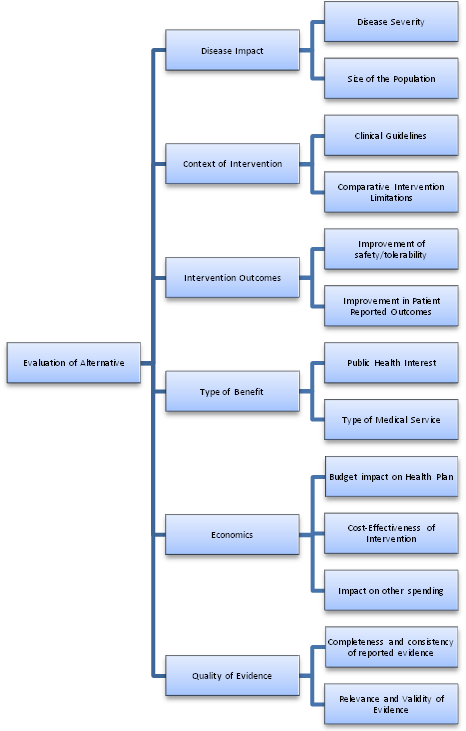


Decision tree without Hierarchy Decision Tree with Hierarchy
